# B Cells and B Cell Depletion in Autoimmunity and Atherosclerosis

**DOI:** 10.3390/life16060923

**Published:** 2026-05-30

**Authors:** Jenny Lue Solomon, Anjali Dubbaka, Ankita Srivastava, Elise Belilos, Joshua De Leon, Steven E. Carsons, Allison B. Reiss

**Affiliations:** 1 Department of Medicine, NYU Grossman Long Island School of Medicine, Mineola, NY 11501, USA; jenny.lue@nyulangone.org (J.L.S.); anjali.dubbaka@nyulangone.org (A.D.); elise.belilos@nyulangone.org (E.B.); joshua.deleon@nyulangone.org (J.D.L.); steven.carsons@nyulangone.org (S.E.C.); 2 Department of Foundations of Medicine, NYU Grossman Long Island School of Medicine, Mineola, NY 11501, USA; ankita.srivastava@nyulangone.org

**Keywords:** B cells, cardiovascular risk, atherosclerosis, autoimmune disorder, FcRn antagonists, B cell activating factor, treatment

## Abstract

Although previously B cells had been underestimated in comparison to T cells in their role in autoimmunity, now, their impact is well established. Via secretion of autoantibodies, presentation of autoantigens, regulation of antigen processing and presentation, and release of inflammatory cytokines, B cells can mediate cytotoxicity and lead to organ damage. B cell depletion via CD20 targeting effectively eliminates B cells in the blood and primary lymph organs and has found an effective role in the treatment of both rheumatological and neurological diseases. The neonatal Fc receptor (FcRn) is a key component of immune regulation that prevents IgG (produced by B cells) from degradation by lysosomes, sending it back into the extracellular compartment, thereby extending its half-life. This abundance of pathogenic IgG can lead to the development of autoimmune disease. The interplay between these two mechanisms of autoimmunity provides the great potential for combination therapy to reduce existent pathogenic IgG as well as prevent the production of new autoantibodies, though further investigation is needed to determine the risks, particularly of infection. This paper will explore existing B cell depleting treatments and FcRn inhibitors, and consider the potential impact for autoimmune disease as well as for the treatment of atherosclerosis.

## 1. Introduction

The human immune system employs inflammation as an important early response by triggering mechanisms that neutralize pathogens, repair damage, and lead to eventual healing. However, when inflammation is driven by autoimmune responses and the immune system erroneously targets self-antigens, tissue damage and adverse health consequences can result. B lymphocytes are key players in this process, and they participate via antigen presentation, cytokine secretion, and immune modulation. Working in parallel to this mechanism, the neonatal Fc receptor specifically preserves immunoglobulin G by diverting this antibody class from degradation by lysosomes. The dysregulation of B cell and neonatal Fc receptor function can amplify pathogenic autoantibodies and set off a cascade of disorders that drive multiple rheumatologic and neurologic conditions [1]. Furthermore, atherosclerosis is now understood to be both a disease of excess arterial lipid accumulation and an inflammatory disorder [2]. Different types of B cells have demonstrated divergent effects, both protective and deleterious, on the development of atherosclerosis. Subsequently, as a growing body of literature has shown, B cell inhibition and neonatal Fc receptor blockade can effectively treat some autoimmune and inflammatory diseases, with strong potential for further novel applications. This review examines current and cutting-edge B cell-depleting therapies and FcRn inhibitors, evaluating their potential application in managing autoimmune and inflammatory diseases, including atherosclerosis.

## 2. B Cell Function in the Immune System

The human immune system has evolved to recognize and destroy foreign invaders such as viruses, bacteria, fungi, and other organisms via two mechanisms: natural and adaptive immune systems [3]. Natural immunity consists of the complement system, macrophages, and natural killer (NK) cells, while adaptive immunity is made up of the response to antigens by B cells and T cells [4]. Blood cells are divided into myeloid and lymphoid lineages. Myeloid cells are responsible for innate immunity, whereas lymphoid cells are responsible for adaptive immunity. B cells are lymphocytes whose progenitors are formed in the bone marrow, and they play a pivotal role in the adaptive immune system through antibody production and upregulation of adaptive responses [5].

B cells are formed from hematopoietic stem cells and require differentiation and maturation to allow them to interact with antigens and produce antibodies [6]. It is via this process that the B cells perform the crucial functions of the adaptive immune system: recognizing self-antigens from non-self or foreign antigens and creating the memory of previous encounters with those antigens so that when exposed to them in the future, the body may effect a more rapid response [7,8]. B cells can be found in lymph nodes, specifically the cortex, and it is there that they make up B cell follicles: primary follicles with naive B cells and secondary follicles that have germinal centers. In these germinal centers, B cells activate, multiply, and differentiate into plasma cells or memory B cells [9]. B cells can also be found in the white pulp of the spleen, in the tonsils, and Peyer’s patches. The B cell receptor recognizes each unique antigen and activates naive B cells (which express IgM and IgD) to undergo germinal center reaction so that B cells recognizing that specific B cell receptor clonally expand and quickly proliferate to become CD27+ memory B cells [10]. The adaptive immune system relies on the memories generated following the initial response to an antigen, which is described as the primary response [11]. B lymphocytes express cell surface immunoglobulin receptors that can recognize different antigen epitopes. This is also the process by which autoimmunity can develop, when the same immune response is galvanized against self- or altered self-antigens. Exposure to these self-antigens, as seen in systemic lupus erythematosus (SLE), Sjogren’s disease, or rheumatoid arthritis (RA), promotes autoantibody production by B cells resulting in the recruitment of proinflammatory cytokines [12].

Via immunoglobulin class switching, B cells can express other antibody isotypes such as IgG, IgA, or IgE. These class-switched B cells are frequently present in autoimmune neurological conditions [10]. Each antibody isotype (IgA, IgD, IgE, IgG, IgM) binds to its corresponding unique Fc receptor on innate immune cells, and these antibodies bind to target antigens to recruit Fc receptor-mediated target cell destruction with NK cells, macrophages, and neutrophils [13]. Furthermore, B cells can also express co-stimulatory molecules and pro-inflammatory cytokines like interleukin-6 and tumor necrosis factor, which then activate interferon-γ secreting type 1 T helper cells. Conversely, some B cells, referred to as B regulatory cells, can also produce anti-inflammatory cytokines like IL-10 [14]. CD 19 and CD 20 are expressed in all B cell lineages except plasma cells; these are proteins found on B cell surfaces that help to regulate cell activation and differentiation and are a target for therapies for both neurological and rheumatological diseases discussed later in this paper [15].

## 3. The Role of the Neonatal Fc Receptor

The neonatal Fc receptor (FcRn) is a major histocompatibility complex class I (MHC I) related molecule that binds IgG and albumin in a pH-dependent manner [16,17]. FcRn protects IgG from degradation and enables it to cross barriers like the placenta to provide fetal humoral immunity passed from mothers [18]. It is made up of three extracellular alpha domains and a transmembrane domain. Previously, it was assumed that FcRn was only expressed on neonatal cells; however, now it is understood that FcRn regulates IgG function by diverting the formed immune complexes to activate CD8+ and CD4+ T cells, which leads to increased B cell response and IgG production [19]. This response is noted in autoimmune diseases such as rheumatoid arthritis and inflammatory bowel disease [20,21]. IgG is the most commonly found immunoglobulin in serum because it has the longest half-life due to this recycling process performed by FcRn; it is also associated with many autoimmune diseases.

Both pathogenic IgG and normal IgG are salvaged indiscriminately via the FcRn [22]. FcRn binds to IgG in placental syncytiotrophoblasts and crosses the stroma through the fetal endothelium into fetal circulation [23]. In this way, maternal antibodies induced by vaccination can be transferred to the fetus and maternal autoantibodies can cause fetal heart block in Sjogren’s syndrome and lupus [24,25]. It is also the mechanism by which B cell inhibitors such as rituximab can cause hypogammaglobulinemia in neonates [20,26]. Blockade of FcRn enhances IgG degradation and reduces IgG serum concentration; therefore, FcRn inhibitors such as nipocalimab can be used for treating myasthenia gravis (MG), where pathogenic IgG autoantibodies targeting acetylcholine receptors alter neuromuscular transmission preventing acetylcholine from binding and leading in the muscle weakness seen in MG [27,28].

The mechanism of FcRn upregulation occurs via several pathways; the most well understood is activation of nuclear factor kappa-light-chain-enhancer of activated B cells (NF-κB) via tumor necrosis factor-α (TNF-α) stimulating Fc fragment of IgG receptor and transporter (FCGRT) gene transcription, which then increases FcRn protein in immune cells such as macrophages and monocytes [29]. Toll-like receptor (TLR) ligands such as lipopolysaccharides bind to the TLR and set off a signaling cascade that upregulates FcRn transcription factor NF-κB, which increases the transcription of FcRn, enhancing FcRn production, which, in turn, increases IgG recycling [29]. Alternatively, TGF-β1 can increase FcRn by activating c-Jun N-terminal kinase (JNK) binding to the FCGRT gene promotor, which not only increases the expression of FcRn but also enhances IgG transcytosis [30]. There are also populations homozygous for the variable number of tandem repeat (VNT) 3 allele which promotes transcription of the promoter part of FGCRT so these individuals make more FcRn protein for binding to IgG and thus can recycle antibodies more effectively than heterozygous individuals [20]. Conversely, downregulation of FcRn is mainly via the Janus kinase (JAK) and signal transducer and activator of transcription (STAT) pathway. STAT-1 binds to the interferon-γ (IFN-γ) activation site in the FCGRT promoter region and suppresses transcription [29]. Glucocorticoid downregulation of FGCRT transcription has been shown to occur in mouse models via a reduction in the expression of the FcRn α-subunit, implying hormonal regulation of FcRn [20]. Lastly, FcRn inhibitors do not affect transcription of FGCRT; however, they functionally block the interaction between IgG and FcRn, preventing the recycling of IgG and leading to catabolism, the clinical applications of which will be elucidated later in this paper. Table 1 summarizes the effects of key regulatory pathways on FcRn.

## 4. Autoimmunity and Atherosclerosis

The pathogenesis of atherosclerosis is now understood to be a chronic inflammatory disease that results in the formation of atheromas in arteries [31]. Multiple studies have established this connection, especially with the recognition that patients with inflammatory diseases, such as rheumatoid arthritis or systemic lupus erythematosus, have premature atherosclerosis without the traditional risk factors such as diabetes mellitus, smoking history, or obesity [32,33]. Prior clinical studies have revealed a positive association between stroke risk and the presence of activated CD19+ and CD 86+ B cells and a negative association with the presence of unswitched memory B cells [34,35,36]. B cells have been shown in in vitro and animal studies to promote the development of atheroma via differentiation into plasma cells that produce pathogenic IgG that activates the inflammasome by directly increasing toll like receptor 4 (TLR4) activity, which is especially notable as TLR4 is found in unstable plaques, indicating that it plays a major role in coronary artery disease progression [37,38]. Mouse models have demonstrated that the loss of macrophage FcRn could halt the propagation of pathogenic plaque IgG, leading to decreased lesion size, necrosis, and inflammation overall [39]. This has prompted the idea that targeting FcRn could promote the efflux of cholesterol and prevent atherosclerosis.

## 5. B Cell Function in Rheumatological Disease

B cells have been shown to have multiple roles in rheumatoid arthritis, SLE, Sjogren’s syndrome, and IgG4 related disease. In rheumatoid arthritis, B cells are involved in antigen presentation, cytokine secretion, autoantibody production, and the formation of ectopic lymphoid structures in the synovium [40]. B cells serve as antigen-presenting cells in RA, presenting their own antigens to CD4+ T helper cells, which are significantly increased in synovium and peripheral blood in RA [41]. B cells also produce autoantibodies, including rheumatoid factors and anti-citrullinated protein antibodies, which are associated with disease activity and severity. Additionally, B cells secrete proinflammatory cytokines and chemokines, including IL-6 and TNFα, further amplifying the inflammatory cascade and promoting joint destruction [42]. In SLE, B cells are responsible for generating a wide array of pathogenic autoantibodies including anti-dsDNA and anti-Smith, which form immune complexes and mediate tissue injury [43]. Dysregulated B cell tolerance checkpoints allow autoreactive B cells to persist in SLE [44,45]. In SLE, type I interferon signaling promotes the differentiation of polyreactive naive B cells into memory B cells, rather than keeping them in a naive state as in healthy individuals [46]. Among these memory B cells, those expressing the immunoglobulin heavy variable gene (IGHV) 4-34 are especially expanded. The frequency of unswitched memory B cells expressing IGHV4-34 has been strongly correlated with SLE disease activity, as measured by the SLE Disease Activity Index (SLEDAI) [47]. In Sjogren’s syndrome, B cells play similar roles, including antigen presentation, cytokine production, and autoantibody production (anti-SSA/Ro, anti-SSB/La) [48]. In addition, B cells form ectopic germinal centers within exocrine glands, increase plasmablasts and plasma cells, dysregulate B cell survival factors and regulatory cells, and increase risk of lymphomagenesis by continuous stimulation of autoreactive B cells by immune complexes [49,50]. In IgG4-related disease, B cells drive disease by presenting antigens to T cells, undergoing class switching via CD4+ T follicular helper cells (specifically, the Tfh2 subset) to produce IgG4, and differentiating into plasmablasts and plasma cells that mediate inflammation and fibrosis [51,52].

## 6. B Cell Function in Neurological Disease

Although early models of multiple sclerosis emphasized pathogenic T cells as the primary drivers of disease, it is now clear that B cells and humoral immunity also play a major role [53,54]. Diagnostic criteria for multiple sclerosis now include increased intrathecal IgG synthesis and CSF oligoclonal bands or kappa free light chains, indicating the role of the humoral response in the disease process [55,56,57]. In multiple sclerosis, B cells are not primarily pathogenic through antibody secretion as in other autoimmune diseases, but rather through their ability to act as potent antigen-presenting cells, secreting proinflammatory cytokines, activating autoreactive T cells, and amplifying CNS inflammation [58,59]. Memory B cells cross the blood–brain barrier and are thought to be reactivated within the CNS, where they undergo antigen-driven affinity maturation, clonal expansion, and ultimately differentiate into antibody-producing plasma cells [60,61,62,63]. However, it has been shown that patients who improve with B cell depletion therapy often do so without a correlating decrease in autoantibody levels, indicating that B cells must contribute to autoimmune disease through antibody-independent mechanisms, likely related to cytokine production [64,65,66].

B cells also play a central and multifaceted role in the immunopathogenesis of myasthenia gravis [67]. They are the source of pathogenic autoantibodies against targets such as Acetylcholine Receptor (AChR) and Muscle-specific tyrosine kinase (MuSK), which disrupt neuromuscular transmission [68,69]. In the thymus—particularly in patients with thymic follicular hyperplasia—B cells form ectopic germinal centers where they undergo somatic hypermutation, antigen-driven clonal expansion, and differentiation into plasma cells that secrete autoantibodies [70,71,72]. These B cells also produce pro-survival signals; for example, B cell Activating Factor (BAFF) supports the survival of class-switched B cells within the thymus, enabling the persistence of autoreactive clones [73,74]. Memory B cells are especially important in disease relapse, as they re-emerge after B cell depletion therapy and may repopulate pathogenic clones [75,76,77].

B cells play a critical pathogenic role in neuromyelitis optica spectrum disorder (NMOSD), not only by producing aquaporin (AQP)4 IgG autoantibodies but also through antigen presentation, cytokine secretion, and germinal centers [78]. In NMOSD patients, there is a selective expansion of CD19int CD27high CD38high CD180- plasmablast-like B cells in the peripheral blood, which secrete AQP4-IgG in response to IL-6 stimulation [79,80,81,82]. Transcriptomic profiling of B cell subsets from NMOSD patients has shown skewing toward an antibody-secreting cell phenotype, driven in part by upregulated IL-2 signaling, especially in naive B cells expressing CD25 [82,83]. Furthermore, B cells in NMOSD can act as antigen-presenting cells, supporting T follicular helper-like responses and isotype switching, which sustains the humoral autoimmune loop [80,84]. Germinal center activity persists in lymphoid tissues of NMOSD patients, and deep cervical lymph node aspirates have revealed intranodal AQP4-IgG synthesis; this activity has been shown to be dramatically reduced by B cell depletion therapy [85]. Finally, there is evidence that reduced interferon gamma signaling facilitates IL 6 upregulation in B cells, thereby exacerbating the inflammatory Th17 response in NMOSD [86].

## 7. The Role of B Cell Depletion as Therapy

B cells are pivotal in the pathogenesis of autoimmune diseases, making therapeutic strategies that target and deplete B cells a cornerstone of treatment. The most widely studied and used B cell depletion therapy is rituximab, an anti-CD20 chimeric monoclonal antibody [87]. Rituximab targets CD20 expressed on pre-B cells through memory B cells, but not on long-lived plasma cells, allowing selective depletion of B cell subsets while sparing most antibody-secreting plasma cells [88]. After binding CD20, rituximab induces B cell elimination through antibody-dependent cellular cytotoxicity, complement-dependent cytotoxicity, apoptosis, and antibody-dependent phagocytosis [89]. Its therapeutic effects even extend beyond depletion of autoantibody-producing precursors, as rituximab also removes B cells that act as antigen-presenting cells and secrete pro-inflammatory cytokines, thereby reducing T-cell activation and inflammatory amplification [90]. Rituximab elicits a clinical response across numerous autoimmune diseases, often occurring before major reductions in circulating autoantibody levels, supporting the concept that antibody-independent B cell functions are central to disease activity [91]. Following rituximab therapy, heterogeneous patterns of B cell repopulation have been shown to be influenced by disease type and the patient’s individual immune profile, which may guide personalized retreatment strategies [92,93].

Obinutuzumab is a humanized glycoengineered type II anti-CD20 monoclonal antibody that induces more potent B cell depletion than earlier agents through enhanced antibody-dependent cellular cytotoxicity and direct cell death, with less effect on complement-mediated lysis [94,95]. In vitro studies using blood from patients with rheumatoid arthritis and systemic lupus erythematosus have shown that obinutuzumab causes at least two-fold greater B cell cytotoxicity than rituximab, especially via Fc gamma receptor-mediated mechanisms, likely due to better interaction with immune effector cells and less internalization of CD20 [96,97]. In murine models of SLE, obinutuzumab achieved more complete B cell depletion in diseased mice, reduced autoantibody titers, mitigated glomerulonephritis, and prolonged survival more effectively than rituximab [98]. Clinically, the randomized, double-blind phase-2 NOBILITY trial in proliferative lupus nephritis showed that adding obinutuzumab to standard therapy enhanced renal response rates at both 52 and 104 weeks, with no unexpected safety concerns [99].

Obinutuzumab has also shown promise in treatment-resistant ANCA-associated vasculitis, where lower dose regimens achieved remission in patients who had failed or relapsed after rituximab, while maintaining good tolerability and durable B cell depletion [100]. Furthermore, in a humanized mouse model of multiple sclerosis, obinutuzumab treatment reduced spinal cord pathology, showing potential utility in B cell-driven neuroinflammatory conditions [101,102].

Although ocrelizumab and ofatumumab are both humanized anti-CD20 monoclonal antibodies that deplete CD20+ B cells and have been approved for use in multiple sclerosis, they differ in structure, route of administration, and effector mechanisms [103,104,105]. Ocrelizumab is a humanized IgG1 anti-CD20 antibody given by intravenous infusion and has been approved for relapsing-remitting multiple sclerosis and primary progressive multiple sclerosis after the OPERA I/II and ORATORIO phase-3 programs demonstrated reduced relapse activity and slower disability progression for primary progressive multiple sclerosis [106,107]. Mechanistically, ocrelizumab depletes B cells primarily via enhanced antibody-dependent cellular cytotoxicity and Fc-mediated effector functions, disrupting antigen presentation, cytokine production, and autoantibody-precursor pools that drive autoimmune inflammation [108,109]. Compared with rituximab, ocrelizumab demonstrates a distinct effector profile, producing stronger antibody-dependent cell-mediated cytotoxicity while generating less complement-dependent cytotoxicity in vitro, a combination that may translate into more effective modulation of pathogenic B cell responses in vivo [110,111,112]. As a humanized monoclonal antibody, ocrelizumab also carries a lower risk of immunogenicity with repeated dosing, reducing the likelihood of developing human anti-human antibodies compared with the human anti-chimeric antibody responses observed with rituximab, thus potentially offering a more favorable overall benefit–risk profile [113,114].

Ofatumumab is a fully human IgG1 anti-CD20 antibody that binds a distinct, CD20 epitope proximal to the membrane and produces potent B cell lysis through both complement-dependent cytotoxicity and antibody-dependent cell-mediated cytotoxicity [115]. It is formulated for subcutaneous self-administration (20 mg induction weekly ×3 then monthly) and has been approved for relapsing forms of multiple sclerosis following the ASCLEPIOS I and II trials, which showed significant reductions in the annualized relapse rate versus teriflunomide [116].

Clinically, both ocrelizumab and ofatumumab produce rapid and profound peripheral B cell depletion with reductions in MRI activity and multiple sclerosis relapses, but they differ in practical aspects (IV infusion monitoring and dosing schedule for ocrelizumab versus at-home monthly subcutaneous dosing for ofatumumab) and in their safety/real-world profiles [117,118]. Choosing among antiCD20 biologics is often guided by disease phenotype, patient preference regarding route and monitoring, prior immunogenicity to chimeric antibodies, and emerging comparative effectiveness and safety data from randomized trials and observational cohorts [119,120].

**Figure 1:**
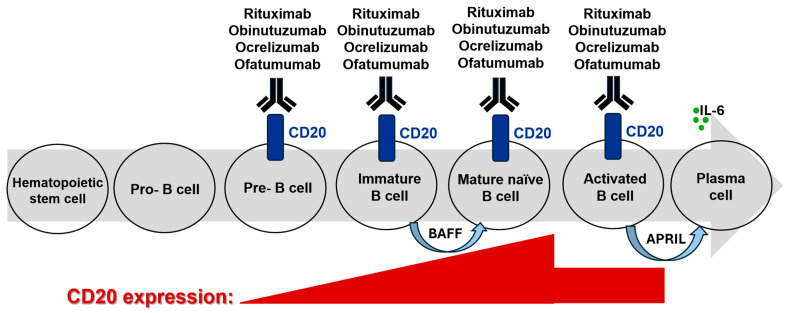
The role of anti-CD20 therapies in the B cell development pathway. This type of therapy selectively depletes CD20-positive cells while sparing long-lived plasma cells, and thus immunoglobulin levels do not immediately fall to zero with anti-CD20 antibody-induced B cell depletion. B-cell-activating factor (BAFF) fosters B cell survival, maturation, and differentiation. A proliferation-inducing ligand (APRIL) facilitates class-switch recombination, drives maturation of plasmablasts into plasma cells and promotes plasma cell survival. Interleukin (IL)-6 drives activated B cells to differentiate into antibody-secreting plasma cells while also promoting their survival and expansion. Collectively, these cytokines form interconnected, reinforcing loops that sustain B cell activation and survival.

Table 2 provides a comparison of key properties of antibodies targeting CD20 in current clinical use. Figure 1 depicts the depletion of CD20-positive B cells across much of the B cell lineage with anti-CD20 therapies.

Inebilizumab is a humanized, afucosylated IgG1 monoclonal antibody that binds to CD19, a B cell surface antigen expressed on a broad range of B-lineage cells—including plasmablasts and some plasma cells—thus enabling more comprehensive B cell depletion than earlier CD20-targeted therapies [121]. By engaging Fc gamma receptors on effector cells, inebilizumab induces B cell depletion primarily through antibody-dependent cellular cytotoxicity and cellular phagocytosis. In contrast to the anti-CD20 antibody rituximab, inebilizumab does not rely on complement-dependent cytotoxicity [122]. In preclinical studies, inebilizumab showed potent depletion of CD19+ B cells, including early progenitors and plasma cells, as well as superior activity compared to anti-CD20 antibodies in achieving and sustaining B cell depletion in bone marrow [123]. Clinically, in the phase II/III N-MOmentum trial in AQP4-IgG-positive neuromyelitis optica spectrum disorder (NMOSD), inebilizumab treatment led to rapid, durable B cell and plasmablast/plasma cell depletion, a 77% reduction in adjudicated NMOSD attacks, and long-term efficacy in the open-label extension without new safety signals [124,125]. Exploratory analyses of N-MOmentum also demonstrated that deeper B cell depletion after the initial dosing correlated with sustained clinical benefit, including lower annualized attack rates and reduced MRI lesion formation [126]. Importantly, the engineering of inebilizumab for enhanced affinity to Fc gamma R3A via afucosylation translates into strong effector activity even in individuals with less favorable Fc receptor polymorphisms, ensuring efficacy across genotypes [127]. Similar to anti-CD20 antibody therapy, immunoglobulin levels should be monitored during inebilizumab therapy, as sustained B cell depletion can lead to reductions in IgG and IgM over time, thus increasing infection risk [128,129].

The greatest risks of all B cell depleting therapies are a consequence of precisely why they are so effective in autoimmune disease: immunosuppression. The risks of these severe adverse effects of B cell depletion vary with the underlying disease, concomitant use of steroids or other immunosuppression, and the duration of treatment. Severe infection and hypogammaglobulinemia are significant risks. Older patients (50 and over), lower baseline IgG prior to exposure, malignant indications for use, and concomitant steroid use are all risk factors for hypogammaglobulinemia [130]. Furthermore, the risk of COVID-19 for a large cohort of oncology patients on B cell depleting therapy showed 6- to 7-fold increase in the risk of hospitalization or death [131]. Another known risk of B cell depletion is the reactivation of the Hepatitis B virus, with an anticipated incidence of >10% especially in the setting of high dose glucocorticoids (at doses of at least 20 mg prednisone for four or more weeks) [132]. Pneumocystis jiroveccii pneumonia (PJP) is another potential risk for patients receiving B cell depletion therapy—in healthy adults the risk of developing PJP is virtually zero; however, previous studies of patients on rituximab have shown an incidence of up to 2.1% [133]. The use of prophylaxis with trimethoprim-sulfamethoxazole significantly reduces the risk of mortality for patients receiving rituximab, with an incidence hazard ratio of 0.2 (95% CI, 0.10–0.42) [134]. Though a rare adverse effect, the development of progressive multifocal leukoencephalopathy (PML), an often-fatal demyelinating disease caused by the John Cunningham virus, can occur with 2.56 confirmed PML cases per 100,000 cases for patients on rituximab [135]. Table 3 highlights the adverse effects associated with B cell depletion induced by specific treatments.

Chimeric antigen receptor (CAR) T cell therapy represents a new frontier for B cell depletion in which the application of B-cell-specific CD19 can profoundly deplete or eliminate CD19-expressing B cells. This would, theoretically, use the patient’s own engineered cells to “reset” autoimmune disease and has the potential to be curative [143]. While clinical experience is in the early stages, the process involves taking a patient’s leukocytes, transfecting them with a lentiviral vector encoding CAR that targets the CD19 surface molecule, allowing in vitro expansion, followed by reinfusion back into the patient along with lymphodepleting chemotherapy such as fludarabine and cyclophosphamide in order to create the ideal environment for these modified T cells to survive and replicate [144]. A small study comparing rituximab-treated patients versus those treated with CAR T cells showed complete B cell depletion in lymph tissue with CAR T therapy, whereas in the rituximab group even though patients had absent peripheral B cells, they still retained B cells in their lymph tissue [145]. Furthermore, in a case series of fifteen patients with SLE, inflammatory myositis, or systemic sclerosis who received one infusion of CD19 CAR T cells, all achieved remission to the point of discontinuing all other immunosuppressive therapy during the 15-month follow up, with relatively few adverse events [146]. The CAR T cell approach has also been discussed for various neurological diseases such as myasthenia gravis, multiple sclerosis, and neuromyelitis optica spectrum disorder. Although there have been rare reports of T cell lymphoma after CAR T cell therapy, there has not been substantial evidence of cases of malignant transformation due to CAR T cell therapy and in one case, the existence of the malignant T cell line pre-dated the CAR T cell therapy [147,148]. Ultimately, further research is necessary, especially larger clinical trials and long-term follow up, to determine the efficacy of this potentially curative treatment.

## 8. B Cell-Targeted Therapies in Atherosclerotic Heart Disease

B cell depletion has been shown to alter the development of atherosclerosis in mouse models. The role of B cells in cardiovascular disease has been well established; the presence of B cells has been demonstrated in infarcted cardiac tissue from the first day of infarction to a week later [149]. The specific lineage of B cell depleted can determine whether the outcome is pro-atherogenic or anti-atherogenic [150]. In one study examining plaque development in mice with and without B cell depletion, mice were treated every 3 weeks with a validated mouse monoclonal CD20 antibody and, although plasma cholesterol levels were not affected, the antibody treatment led to a significant reduction in atherosclerotic lesions [151]. The results were found in both apolipoprotein E–deficient and low-density lipoprotein (LDL) receptor-deficient murine atherosclerosis models. In this same study, there was a notable reduction in proatherogenic IFN-γ in CD20 monoclonal antibody-treated mice compared to untreated controls [151]. In another study, B cell depletion by an anti-CD20 antibody prevented the development of atherosclerosis and stopped the progression of existing plaques in mice, despite not affecting the hyperlipidemia brought on by a high-fat diet. The mechanism of atheroprotection was specific to the depletion of B2 B lineage cells. This was supported by increased atherosclerosis when B2 B cells were transplanted into B-cell-depleted apolipoprotein E-deficient mice [152]. The deleterious impact of B cells in atherosclerosis was shown in C57/BL6 mice after myocardial infarction induced by left coronary ligation [153]. B cell infiltration into the ischemic cardiac tissue was greatly reduced with CD20 antibody treatment, and this reduction in B cells was accompanied by a smaller infarct size, better heart function and lower levels of myocardial inflammation. These effects were attributed to lower monocyte mobilization and recruitment when B cells are scarce, leading to the theory that B cell depletion could potentially have applications in preventing a pathogenic monocyte response after myocardial infarction.

The conflicting effects of B cells on cardiovascular disease may be attributed to B cell heterogeneity. B1 cells are thought to be atheroprotective, which depends on IgM secretion, but B2 cells are proatherogenic due to pathogenic IgG antibodies against oxidized low-density lipoprotein [154]. Meeuwsen et al. found that higher levels of unswitched memory B cells were independently associated with protection against cardiovascular events. These unswitched memory cells expressed more IgM on the surface of the cells, although it was not clear if the amount of IgM expressed from unswitched memory cells actually produced higher serum IgM levels [154]. The regulatory B cell subset of specialized B lymphocytes has an immunomodulatory effect [155]. In murine myocardial infarction models, the transfer of Bregs actually improved ventricular remodeling and cardiac function, likely through the mechanism of reduced expression of C-C motif chemokine receptor 2 in monocytes which blocks proinflammatory monocyte proliferation in the myocardium [156]. Treatment with an IL-10 antibody reversed the protection of Bregs in the myocardium, thereby promoting inflammation and delaying healing. Wu et al. generated mice with B-cell-specific deletion of IL-10, and found that the loss of function of this cytokine exacerbated damage from acute myocardial infarction induced by surgical ligation of the left anterior descending coronary artery [157]. In these IL-10-deficient mice, the outcome after myocardial infarction led to reduced cardiac function and prolonged inflammation in the tissue compared to mice with intact IL-10 expression. Wu et al. also found that IL-10-producing B cells in pericardial adipose tissues infiltrate the myocardium and play a role in healing after acute myocardial infarction in mice. The B cells from pericardial adipose tissue had a surface phenotype consistent with CD5-positive B cells, enriched in the cytokine IL-33 and the chemokine CXCL13. Heinrichs et al. induced myocardial infarction in mice by ligating the left anterior descending coronary artery and, by neutralizing CXCL13, they discovered that CXCL13 mediates selective B cell influx to the damaged myocardium [158]. This particular B cell subset expresses the receptor for CXCL13 and they act in the myocardium as a key source of myocardial TGF-ß1. TGF-ß1 may support scar formation and control inflammation early after myocardial infarction but can lead to stiffening and fibrosis if production is too zealous. These studies highlight the complexity of the role of B cells in cardiovascular disease. Total B cell depletion would take away the atheroprotective function of B1 and Breg cells alongside the deleterious B2 cells [159].

Anti-BAFF has also been shown to decrease atherosclerosis in mouse models. An anti-BAFF blocks the signals that BAFF sends to B cells by binding to BAFF receptors to stop B cell maturation and proliferation. This ultimately reduces autoantibody production and, by depleting B2 cells while sparing B1a cells, can be particularly atheroprotective [160,161]. Unexpectedly, BAFF neutralization has been shown to create larger atherosclerotic plaques, expand macrophage presence in lesions, and increase inflammatory markers despite the B2 cell depletion [162]. However, alternative BAFF-binding receptor, transmembrane activators and calcium modulators and cyclophilin (TACI) ligand interactors in myeloid cells can reduce atherosclerosis. The net effect of BAFF modulation on atherosclerosis therefore depends on the balance of B cell effects and B-cell-independent anti-inflammatory actions on myeloid cells mediated through TACI.

Patients with SLE have an increased risk of cardiovascular disease and an increased prevalence of atherosclerotic plaque as compared to the general population as they have the burden of a pro-inflammatory state, cytokines, autoantibodies in addition to traditional risk factors (such as diabetes and obesity). While deaths directly from SLE have decreased over time due to better treatment options, mortality in SLE patients due to cardiovascular disease has not. Vascular injury is readily induced by monocytes that take up oxidized LDL and become foam cells, forming fatty streaks; immune complexes invite adhesion molecules to bind to endothelial surfaces and upregulate activated platelets to release IL-1β [163]. This excess cardiovascular risk in patients with autoimmune disease indicates that it will be especially important to determine treatable targets in chronic inflammation to potentially reduce mortality. Matsunaga et al. proposed a framework to identify actionable therapeutic targets in chronic obstructive pulmonary disease and, similarly, this framework can be applied to the chronic inflammation of atherosclerotic disease [164]. The actionable therapeutic targets include earlier screening of patients with autoimmune disease, intensive lipid lowering with agents such as statins, improving high-density lipoprotein (HDL) function with apolipoprotein or HDL mimetics and a strong reduction in systemic inflammation with biologics and other disease-modifying medications [165,166]. A study from Dedemadi et al. showed that SLE patients on the anti-BAFF antibody, belimumab, exhibited improved atheroprotective properties of HDL with treatment. The 35 enrolled patients had high SLE disease activity prior to treatment with belimumab and had HDL that showed impaired cholesterol efflux and antioxidant capacity [167]. After 6 months of belimumab add-on therapy, HDL cholesterol efflux and antioxidant capacity in SLE patients were comparable to that of control subjects, although the sample size was small, limiting the power, and the observation time was short, which did not allow for an evaluation of the effect of the improved HDL functionality.

## 9. FcRn Inhibition Therapy

FcRn is an MHC class I-related receptor that not only prolongs the IgG half-life by recycling it but also influences antigen processing and presentation to T cells, thereby contributing to both humoral and cellular autoimmune responses [168]. FcRn inhibitors like efgartigimod, an engineered antibody fragment with high affinity for the FcRn, can reduce pathogenic IgG levels and decrease the presentation of self-antigens, offering a promising therapeutic strategy for IgG-mediated autoimmune diseases such as myasthenia gravis (MG), primary immune thrombocytopenia, and pemphigus [169,170]. In fact, FcRn blockade selectively reduces pathogenic IgG without causing broad immune cell depletion. By modulating both the innate and adaptive immune compartments, efgartigimod normalizes the dysregulated B cell subsets and changes monocyte and T-cell activation states while preserving overall immune homeostasis, suggesting that its clinical benefits are achieved through antibody reduction rather than generalized immunosuppression [171]. FcRn antagonism can be counterproductive for B-cell-depleting therapies like rituximab, as the mechanism is not selective; therefore both pathogenic IgG and therapeutic monoclonal antibodies are cleared [172]. FcRn is distinct in its affinity and avidity for IgG compared with other Fc receptors [173]. The pH-dependent nature of FcRn binding to IgG results in high affinity in the low pH of the endosome and low affinity in the neutral pH of the systemic circulation; as a consequence, the IgG half-life is prolonged and this prolongation can be leveraged to sustain B-cell-depletion by engineering enhanced FcRn affinity into B cell depleting IgG antibodies, keeping them in the circulation [174,175].

FcRn is not just a passive IgG recycling receptor. Rather, it is active in shaping how FcR-expressing immune cells handle antigen processing, and phagocytosis [19,176]. FcRn can modulate dendritic cell maturation, T cell response and immune complex disposition [177,178].

Efgartigimod alfa was the first FcRn antagonist approved for MG, specifically approved for acetylcholine receptor antibody-positive generalized MG in the US and EU, and for all generalized MG regardless of antibody profiles in Japan [179]. In the phase 3 ADAPT trial, treatment with efgartigimod resulted in rapid, clinically meaningful improvements in muscle strength, daily functioning, and quality of life compared with placebo, with benefits often observed within weeks and reproducible across treatment cycles [180]. Long-term extension data suggest these improvements can be maintained with repeated dosing [181]. Efgartigimod was generally well tolerated, with most adverse events being mild to moderate, supporting its role as an effective and targeted alternative to traditional broad immunosuppressive therapies in generalized MG. One case report used sequential treatment of efgartigimod then rituximab, which appeared to sustain B cell depletion by employing both reduction in pathogenic IgG with FcRn inhibition first, then after a washout period, employing B cell depletion to trigger antibody-dependent cell-mediated cytotoxicity an complement-dependent cytotoxicity [182]. Efgartigimod has the most long-term safety data with more than 850 patient-years of follow-up, which found relatively mild to moderate adverse events and severe infection rates were not statistically different from placebo [183]. Furthermore, efgartigimod did not affect albumin or increase LDL cholesterol levels, confirming that it truly was selective for the IgG-binding site of FcRn, separate from the albumin binding site [183]. However, a smaller study of 20 patients found an increased albumin level in 15 cases of patients who received efgartigimod, so further study is needed to elucidate the pharmacological interactions with the FcRn and albumin recycling pathway [184]. The most common adverse events were headache, non-severe COVID-19 infection, and nasopharyngitis [183].

The humanized monoclonal antibody rozanolixizumab is another FcRn inhibitor that accelerates the degradation of pathogenic IgG antibodies by as much as 78.4%, which is comparable to plasma exchange [185]. Rozanolixizumab treatment brings rapid and clinically meaningful improvements in muscle strength, daily functioning, and patient-reported outcomes in adults with acetylcholine receptor or muscle-specific tyrosine kinase antibody-positive generalized MG. The pooled analysis of the Phase 3 MycarinG study alongside two open-label extension studies showed that repeated cycles of rozanolixizumab produced consistent, clinically meaningful improvements in key myasthenia gravis outcome measures including MG Activities of Daily Living (MG-ADL), Myasthenia Gravis Composite (MGC), and Quantitative Myasthenia Gravis (QMG) scores in adults with acetylcholine receptor or muscle-specific tyrosine kinase autoantibody-positive generalized MG. Similar to efgartigimod, improvements were seen across multiple treatment cycles, and rozanolixizumab was generally well tolerated, with most adverse events being mild to moderate and not increasing in frequency with repeated treatment, supporting its use as a therapeutic option in generalized MG [186]. Recently, rozanolixizumab was given to a pregnant patient who had two prior pregnancies complicated by atrioventricular block to prevent cardiac neonatal lupus and the patient delivered an infant with normal echocardiogram and electrocardiogram [187]. This demonstrates the potential for using the blockade of placental IgG transport in pregnancy, which will be studied further with a planned multicenter prospective trial. Rozanolixizumab’s open-label extension evaluated study subjects for a mean treatment duration of 23 weeks without severe or opportunistic infections, no hypersensitivity reactions, and the absence of albumin or lipid abnormalities; the most common adverse events were headache and diarrhea [188]. However, the same study of 20 patients that evaluated albumin in patients who received efgartigimod also studied rozanolixizumab and 100% of the cases (*n* = 5) had decreased albumin levels, which should prompt further study [184].

Batoclimab, a fully human anti-FcRn monoclonal antibody inhibitor, has also shown promise in generalized MG [189]. In China, a phase 3 randomized controlled trial including 131 patients showed sustained improvement in the MG-ADL score with subcutaneous batoclimab compared to placebo, with a favorable safety profile noted as well. In this study, the maximal reduction in serum total IgG with batoclimab was 70.8% at week 6 [190]. After discontinuation of batoclimab, serum total IgG gradually returned to baseline levels within 4 weeks. The reversibility of this effect is clinically important given the increased infection risk associated with prolonged immunosuppression. Notably, FcRn prevents both immunoglobulins and albumin from lysosomal degradation; thus both rozanolixizumab and batoclimab have been associated with reduced albumin, leading to higher rates of peripheral edema [21,191]. In such cases, efgartigimod may be a more suitable alternative as it has less of an effect on albumin and other immunoglobulins [192].

Nipocalimab is a fully human FcRn-blocking monoclonal antibody that has been studied in several disease processes including hemolytic disease of the fetus and newborn, rheumatoid arthritis, MG, Sjogren’s syndrome and lupus [193,194]. In a phase 1, randomized, double-blind, placebo-controlled study, nipocalimab led to dose-dependent reductions in total IgG, largely unaffected by infusion rate which varied from 7.5 to 60 min [195]. These results support the feasibility of shorter infusion durations for nipocalimab in future clinical use while maintaining predictable pharmacokinetic/pharmacodynamic profiles and acceptable tolerability.

Based on the international phase 2 UNITY study, weekly intravenous nipocalimab resulted in live births at or after 32 weeks without the need for intrauterine transfusion in 54% of cases, which is substantially higher than historical benchmarks, and no instances of fetal hydrops were observed [196]. This was attributed to the FcRn-blocking antibody’s effect on reducing maternal alloantibody transfer, IgG levels, and fetal anemia. The findings from this study suggest that nipocalimab may delay or prevent severe fetal anemia and reduce reliance on invasive intrauterine transfusions in this high-risk population. Based on results from the UNITY trial, another global, multicenter, phase 3, randomized, placebo-controlled, and double-blind AZALEA trial is currently underway to further evaluate nipocalimab in pregnancies at risk for severe hemolytic disease of the fetus and newborn [197].

Nipocalimab has also been studied as an FcRn inhibitor treatment in generalized MG. The phase 2 VIVACITY study on nipocalimab in generalized MG patients who had an inadequate response to standard therapy showed that, when each treatment dose group was compared directly to placebo, the difference was not statistically significant in the primary efficacy measure (MG-ADL score at Day 57) [198]. However, there was a significant dose-dependent trend toward improved muscle weakness and daily functioning, thus supporting the need for further evaluation of nipocalimab as a treatment for gMG. Nipocalimab has also been studied in rheumatoid arthritis, with the phase 2a IRIS-RA study including patients with moderate to severe active, seropositive rheumatoid arthritis who had an inadequate response or intolerance to anti-TNF therapy [199]. Although the primary endpoint, the change in the Disease Activity Score in 28 joints using C-reactive protein (DAS28-CRP) at week 12, did not reach statistical significance, patients treated with nipocalimab showed numerically greater improvements in disease activity scores, joint counts, and patient-reported outcomes compared with placebo. Nipocalimab also produced significant, reversible reductions in total IgG, anti-citrullinated protein antibodies (ACPAs), and circulating immune complexes, with greater clinical benefit seen in those with higher baseline ACPA levels. There is also new, promising data for nipocalimab application for Sjogren’s disease. DAHLIAS, a phase 2 double-blind multicenter trial of nipocalimab in patients with moderate-to-severe Sjogren’s disease showed significantly improved clinical disease activity with nipocalimab compared to placebo, with a favorable safety profile noted as well [200]. Nipocalimab in moderate to severe Sjogren’s Disease (DAFFODIL, NCT06741969), a phase 3 trial of nipocalimab versus placebo, is currently in progress and will follow subjects for 48 weeks, with an open-label extension option up to 143 weeks in total [201]. The VIVACITY trial of nipocalimab evaluated the mean percent change in albumin from baseline over a 24-week double-blind phase and showed a decrease in albumin (percent change was –7.2% vs –2.1% in placebo at week 24), although these values were all within normal limits. Mild elevations in total cholesterol (+7.8%), HDL (+7%), and LDL (8.3%) were also detected, although the ratios remained similar to placebo, which is suggestive of limited effects on cardiovascular risk [202]. There were no differences in serious adverse events between the nipocalimab and placebo arms, and the incidence of infection and headache was similar in both groups. Table 4 provides an overview of the features and mechanism of action of FcRn inhibitors.

Table 5 summarizes the key differences in effects on the immune system when comparing anti-CD20, anti-CD19 and FcRn blocking agents.

## 10. Future Directions

The application of FcRn inhibition to the treatment of autoimmune disease offers the advantage of IgG catabolism while avoiding broad immunosuppression and its inherent elevated infection risk [203]. As mentioned above, FcRn blockade is being studied in maternal-fetal medicine for the prevention of neonatal lupus, which is such an important potential application in light of the serious cardiac manifestations, especially considering that SLE commonly affects women of childbearing age [187]. FcRn inhibitors may have important clinical advantages for the management of autoimmune diseases as compared to plasma exchange or IVIG. Plasma exchange directly removes IgG and thus has a rapid onset of action, explaining its preferential use in emergency situations like a myasthenic crises. However, the effects of plasma exchange are of shorter duration than those of FcRn inhibitors. IVIG can clear immune complexes; however, it is nonspecific whereas FcRn is more selective in its targeting [168]. Furthermore, plasma exchange and IVIG are both blood products, which carry a theoretical risk of infection. IVIG also has risks of infusion reaction, thrombosis, and impact on kidney function, whereas FcRn inhibitors have been largely found to have favorable safety profiles in studies of adults [204]. There is also an opportunity for FcRn antagonists to work synergistically as a “downstream clearance” mechanism to dually block pathogenic antibodies in combination with B-cell-depleting therapies or BAFF inhibitors [21].

FcRn may also have a role in dyslipidemia, as a recombinant humanized IgG1 antibody has been found to enhance liver triglyceride homeostasis in experimental models, encouraging macrophages to consume lipids and maintaining reverse cholesterol transport to decrease cholesterol from peripheral cells [205]. By this mechanism, FcRn inhibition may have the opposite of the intended effect of curbing atherosclerosis. Supporting the possible pro-atherosclerotic consequences of FcRn inhibition, LDL and total cholesterol were found to be elevated in study participants receiving nipocalimab, which is theorized to be due to a decrease in serum albumin, a protein that may play a role in regulating cholesterol transport [202,206]. Further study will be needed to see whether patients treated with FcRn inhibition need more careful cardiovascular monitoring despite not having traditional atherosclerotic heart disease risk factors.

Perhaps more promising in terms of atherosclerotic disease is B cell depletion. One study in Korea examined kidney transplant patients who received rituximab; at an 8-year follow-up, those who had received B cell depletion therapy exhibited a significantly lower rate of atherosclerotic cardiovascular disease in comparison to matched controls, although all-cause mortality was unchanged [207]. BAFF inhibition, as mentioned above, may also have the unintended consequence of increased atherosclerosis in SLE patients, and this has been found to be true in mouse models [162,208]. However, further study of highly specific BAFF specific antibodies, rather than entirely neutralizing BAFF, could offer an avenue to deplete B2 cells without impacting B1 cell function. In preclinical models, this approach has shown atheroprotection because B1-derived natural IgM neutralizes oxidized LDL [161,209,210].

Longer-acting forms of FcRn inhibitors, as well as autoinjector and oral versions, may be on the horizon and would facilitate their wider use [211].

## 11. Conclusions

IgG-mediated autoimmune diseases affect multiple organ systems, and the growing body of knowledge regarding the capabilities of two distinct therapeutic approaches: B cell inhibition and FcRn receptor blockade, each individually represents a potential paradigm shift for treatment. B cell depletion with medications such as rituximab and, more recently obinutuzumab has been effective in treating diseases including ANCA vasculitis, rheumatoid arthritis, lupus nephritis, and neuromyelitis optica. FcRn inhibition has now been approved for use in neurological diseases with efgartigimod, rozanolixizumab, and nipocalimab approved to treat generalized myasthenia gravis. Moreover, promising studies investigating the application of this drug class in the realm of rheumatological diseases are ongoing. Exploiting the selection of only IgG and IgG complexes without affecting IgA and IgM avoids broader immunosuppression in these patients. These two distinct yet complementary mechanisms provide clinicians with powerful tools to individualize treatment and minimize risks based on patient characteristics.

## Figures and Tables

**Table 1:** Effect of Regulatory Factors on FcRn.

Regulatory Factor	Effect	Pathway
TNF-α	Upregulation	NF-κB
TLR ligands	Upregulation	NF-κB
TGF-β1	Upregulation	JNK/MAPK
VNTR3	Upregulation	Transcription
IFN-γ	Downregulation	JAK/STAT
Glucocorticoids	Downregulation	Transcription
FcRn inhibitors	Downregulation	Functional Blockade

TNF-α: tumor necrosis factor alpha; NF-κB: nuclear factor kappa-light-chain-enhancer of activated B cells; TLR: toll like receptors; TGF-β1: transforming growth factor beta 1; JNK/MAPK: c-Jun N-terminal kinase/MAP kinase; VNTR3: variable number of tandem repeat 3; IFN-γ: interferon-γ.

**Table 2:** Mechanistic and clinical differentiators across anti-CD20 agents.

Feature	Rituximab	Obinutuzumab	Ocrelizumab	Ofatumumab
Antibody type	Chimeric	Humanized, glycoengineered	Humanized	Fully human
ADCC	Moderate	High	High	Moderate-high
CDC	High	Low	Low-moderate	High
B cell depletion depth	Moderate	High	High	High
Immunogenicity	Higher	Lower	Lower	Lowest
Administration	IV	IV	IV	SC (self-administered)
Key Advantage	Established efficacy	Superior depletion	Favorable safety/immunogenicity	Convenience

ADCC: antibody-dependent cellular cytotoxicity; CDC: complement-dependent cytotoxicity; IV: intravenous; SC: subcutaneous.

**Table 3:** Adverse Effects of B Cell Depletion Therapies.

Adverse Effect	Medication	Incidence	Indication	Reference
Hypogammaglobulinemia	Rituximab	11%	MS	[136]
	Ocrelizumab	12%	MS and NMOSD	[130]
	Rituximab	3.5–4.2%	RA and ANCA vasculitis	[137]
Serious Infection	Ocrelizumab	6%	MS	[136]
	Rituximab	4.4%	RA	[138]
	Ofatumumab	2.9%	MS	[139]
PJP	Rituximab	1.5% (during induction)	ANCA vasculitis	[140]
	Rituximab	1%	NHL	[141]
PML	Rituximab	0.1%	Malignancy, autoimmune disease	[142]

MS: myasthenia gravis; NMOSD: Neuromyelitis optica spectrum disorder; RA: rheumatoid arthritis; ANCA: Anti-Neutrophil Cytoplasmic Antibody; PJP: Pneumocystis jirovecii pneumonia; NHL: Non-Hodgkin Lymphoma; PML: progressive multifocal leukoencephalopathy.

**Table 4:** FcRn inhibitor medications.

Feature	Efgartigimod	Rozanolixizumab	Batoclimab	Nipocalimab
Molecular Structure	Human IgG1 antibody Fc fragment	Humanized anti-FcRn monoclonal antibody with IgG4 Fc domain	Fully human anti-FcRn IgG1 monoclonal antibody	Fully human aglycosylated IgG1 monoclonal antibody
Mechanism of Action	Binds FcRn at the IgG binding site as a high-affinity decoy	Binds FcRn via Fab fragment	Binds FcRn via Fab fragment	Binds FcRn via Fab fragment
FDA Approved Indications	gMG, CIDP	AChR Ab+ or MuSK Ab+ gMG	Not FDA approved	gMG
Potential Applications	ITP, SLE, SPS	ITP, Neonatal SLE	gMG, TED, CIDP	HDFN, SLE, Sjogren’s, RA
Data Source	Human and Murine	Human and Murine	Human and Murine	Human and Murine

gMG: general mysthenia gravis; CIDP: chronic inflammatory demyelinating polyneuropathy; ITP: primary immune thrombocytopenia; SLE: systemic lupus erythematosus; SPS: stiff person syndrome; TED: thyroid eye disease; HDFN: hemolytic disease of the fetus and newborn; RA: rheumatoid arthritis.

**Table 5:** Differentiating among anti-CD20, anti-CD19, and FcRn blockade strategies.

Strategy	Primary Effect	Secondary Effects
Anti-CD20	B cell depletion	↓ APC function, ↓ cytokines, ↓ T-cell activation
Anti-CD19	Broader depletion (including plasma cells)	Greater Ig reduction
FcRn blockade	↓ IgG half-life	Minimal cellular immunosuppression

APC: antigen-presenting cell; Ig: immunoglobulin; IgG: immunoglobulin G; FcRn: neonatal Fc receptor. ↓ = decreases.

## Data Availability

Not applicable.
